# The scaffold protein AXIN1: gene ontology, signal network, and physiological function

**DOI:** 10.1186/s12964-024-01482-4

**Published:** 2024-01-30

**Authors:** Lu Qiu, Yixuan Sun, Haoming Ning, Guanyu Chen, Wenshan Zhao, Yanfeng Gao

**Affiliations:** 1 School of Pharmaceutical Sciences (Shenzhen), Shenzhen Campus of Sun Yat-Sen University, Shenzhen, 518107 China; 2 School of Life Sciences, Zhengzhou University, Zhengzhou, 450001 China

**Keywords:** AXIN1, WNT/β-Catenin signaling, Hippo signaling, TGFβ signaling, AMPK signaling, mTOR signaling, MAPK signaling, Tumorigenesis, Destruction complex, AXIN1-associated phosphokinase complex (AAPC)

## Abstract

AXIN1, has been initially identified as a prominent antagonist within the WNT/β-catenin signaling pathway, and subsequently unveiled its integral involvement across a diverse spectrum of signaling cascades. These encompass the WNT/β-catenin, Hippo, TGFβ, AMPK, mTOR, MAPK, and antioxidant signaling pathways. The versatile engagement of AXIN1 underscores its pivotal role in the modulation of developmental biological signaling, maintenance of metabolic homeostasis, and coordination of cellular stress responses. The multifaceted functionalities of AXIN1 render it as a compelling candidate for targeted intervention in the realms of degenerative pathologies, systemic metabolic disorders, cancer therapeutics, and anti-aging strategies. This review provides an intricate exploration of the mechanisms governing mammalian AXIN1 gene expression and protein turnover since its initial discovery, while also elucidating its significance in the regulation of signaling pathways, tissue development, and carcinogenesis. Furthermore, we have introduced the innovative concept of the AXIN1-Associated Phosphokinase Complex (AAPC), where the scaffold protein AXIN1 assumes a pivotal role in orchestrating site-specific phosphorylation modifications through interactions with various phosphokinases and their respective substrates.

## Introduction

The *AXIN1* gene, originally derived from the *Fused* mouse model, which includes tail bifurcation resulting from the presence of two neural tubes, tail curling due to asymmetric vertebrae closure, and embryonic lethality. This gene assumes a pivotal role in embryonic development by thwarting excessive axial and organ growth [1]. AXIN1 is mostly linked to the WNT/β-catenin signaling pathway. It works with APC, GSK3β, and CK1α to form the destruction complex. This complex controls the phosphorylation modification and ubiquitin-dependent degradation of β-catenin. Remarkably, the scope of the destruction complex extends beyond the confines of WNT/β-catenin signaling. It has become a global regulator of developmental biological signals since it plays a part in the phosphorylation and degradation of entities such as TAZ/YAP [2] and SMADs [3]. Of particular significance, Liu et al*.* reported AXIN1's facilitation of AMPK phosphorylation by LKB1, culminating in the formation of the AXIN1/LKB1/AMPK complex, which exerts inhibitory influence over mTORC1 at the lysosomal surface [4, 5]. These observations underscore the indispensable role played by AXIN1 in orchestrating cellular energy homeostasis and responding to its fluctuations.

AXIN1 plays a well-organized and precise role in complex molecular signaling networks by interacting with certain phosphokinases and the proteins that they bind to. Functioning as a scaffold protein, AXIN1 governs the phosphorylation modifications of a diverse array of substrate proteins mediated by various phosphokinases. Viewing the complex involving AXIN1 and specificity phosphokinases as a unified functional entity provides a more comprehensive understanding of AXIN1's multifaceted molecular and physiological functions. Indeed, AXIN1's involvement in fundamental processes such as cell proliferation, differentiation, maintenance of metabolic homeostasis, and stress response underscores that any deviation or deficiency in its functionality can lead to detrimental outcomes, encompassing a spectrum of conditions, from neurodegenerative disorders to developmental abnormalities in bone structures and tumorigenesis. The amalgamation of challenges and opportunities underscores the promising potential of targeted AXIN1 regulation as a viable strategy for the treatment of various diseases.

Nevertheless, an enduring conundrum persists regarding the precise spatiotemporal attributes of AXIN1's functional roles. Even though AXIN1 plays an important role as a possible drug target at the molecular level, there isn't a lot of comprehensive research on it, especially in the area of applied investigations. Thus, this is a concern for using AXIN1 as a treatment for many disorders. In this comprehensive review, we undertake a systematic and exhaustive examination of the body of research pertaining to mammalian AXIN1 since its inaugural discovery. Within these pages, we present a comprehensive overview of the established molecular functions and physiological roles of AXIN1, spanning diverse domains, including gene expression, protein turnover, protein–protein interactions, signaling networks, physiological functions, and the spectrum of pathological phenotypes.

### Discovery, expression and regulation of AXIN1

#### Expression characteristics and regulation of *AXIN1* gene

**Fig. 1:**
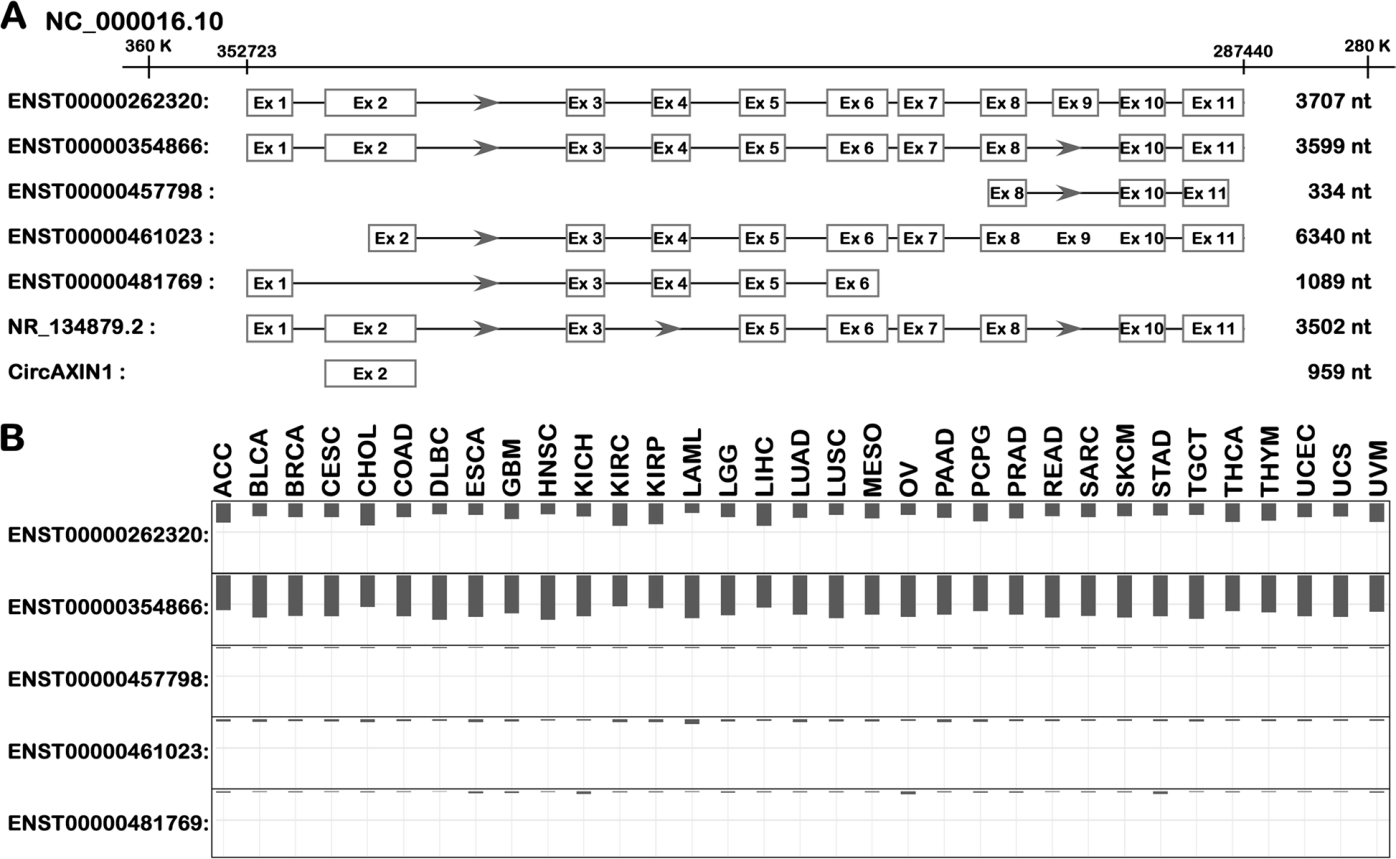
The transcript and expression characteristics of the human *AXIN1* gene. **A** Transcripts and exons of the *AXIN1* gene. **B** The expression preference of five common transcripts of AXIN1 in multiple cancer types. (Data from *Gene Expression Profiling Interactive Analysis*: https://gepia2.cancer-pku.cn/)

The human *AXIN1* gene is located on chromosome 16p13.3 and encompasses 65,284 base pairs, spanning 11 exons and yielding 7 distinct transcripts (NCBI gene number: 8312, reference sequence: NC_000016.10) (Fig. 1, Table 1). In 1997, Zeng Li and colleagues [1] first reported the successful cloning of the *AXIN1* gene in the mouse *fused* locus and its homologous counterpart in humans, sharing an approximate 87% similarity in protein sequences between the two species. *AXIN1* is ubiquitously distributed across both invertebrates and vertebrates, characterized by a high degree of conservation in a range of organisms, including *C. elegans, D. melanogaster, Z. fasciata, Xenopus laevis, Gallus gallus, Mus musculus, Rattus norvegicus*, and humans [1, 6–8].

**Table 1:** Transcript information of human *AXIN1* gene

Transcript ID	RefSeq	Biotype	Longth (nt)	UniProt	Protein	References
ENST00000262320	NM_003502.4	Protein coding	3707	O15169	862aa	[1]
ENST00000354866	NM_181050.3	Protein coding	3599	O15169	826aa	[1]
ENST00000457798	-	Protein coding	334	H0Y830	102aa	[9]
ENST00000461023	-	Retained intron	6340	-	No protein	Ensembl
ENST00000481769	-	Processed transcript	1089	-	No protein	Ensembl
-	NR_134879.2	misc-RNA	3502	-	-	NCBI
-	-	circ-RNA	959	-	295aa	[10]

**Fig. 2:**
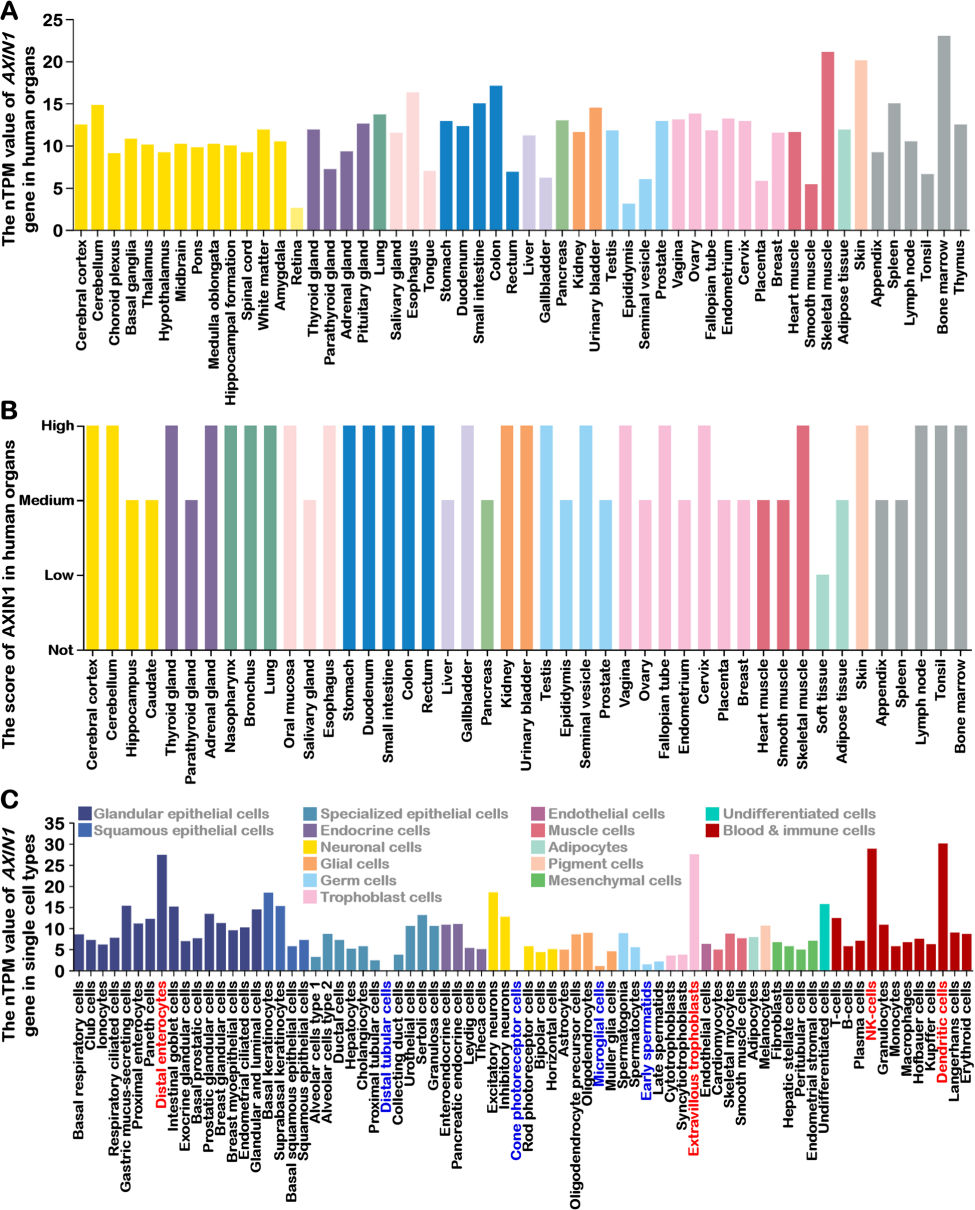
Expression Profile of AXIN1 in Human Tissues. **A** Transcriptional expression levels of the *AXIN1* gene in human tissues. The consensus dataset comprises normalized expression (nTPM) levels for 55 tissue types, created by integrating the HPA and GTEx transcriptomics datasets using an internal normalization pipeline. Color-coding is based on tissue groups, each consisting of tissues with shared functional features. **B** Expression of the AXIN1 protein in 44 human tissues. Color-coding is based on tissue groups, each consisting of tissues with functional features in common. **C** Summary of *AXIN1* expression (nTPM) from all single cell types. Color-coding is based on cell type groups, each consisting of cell types with functional features in common. (Data from *The Human Protein Atlas*: https://www.proteinatlas.org/)

AXIN1 was initially recognized as a critical regulator suppressing axis formation during embryonic development, with its deficiency resulting in axial duplication in *Fused* mice [1]. Nevertheless, it is noteworthy that AXIN1 does not confine its expression exclusively to embryonic development but is broadly expressed in a variety of tissue cells and lacks pronounced tissue specificity (Fig. 2A, B) [11]. This widespread expression profile implies that AXIN1 may possess molecular functions that extend beyond developmental regulation. Notably, single-cell type expression analysis reveals certain distinctive patterns in *AXIN1* expression (Fig. 2C) [12]. For instance, AXIN1 exhibits nearly negligible expression in distal tubular cells and cone photoreceptor cells, while displaying lower expression levels in microglial cells and early spermatids. Conversely, it assumes elevated expression in distal enterocytes, extravillous trophoblasts, natural killer cells, and dendritic cells. The physiological significance of these preferential AXIN1 expression patterns remains enigmatic due to the limited availability of relevant research reports.

**Fig. 3:**
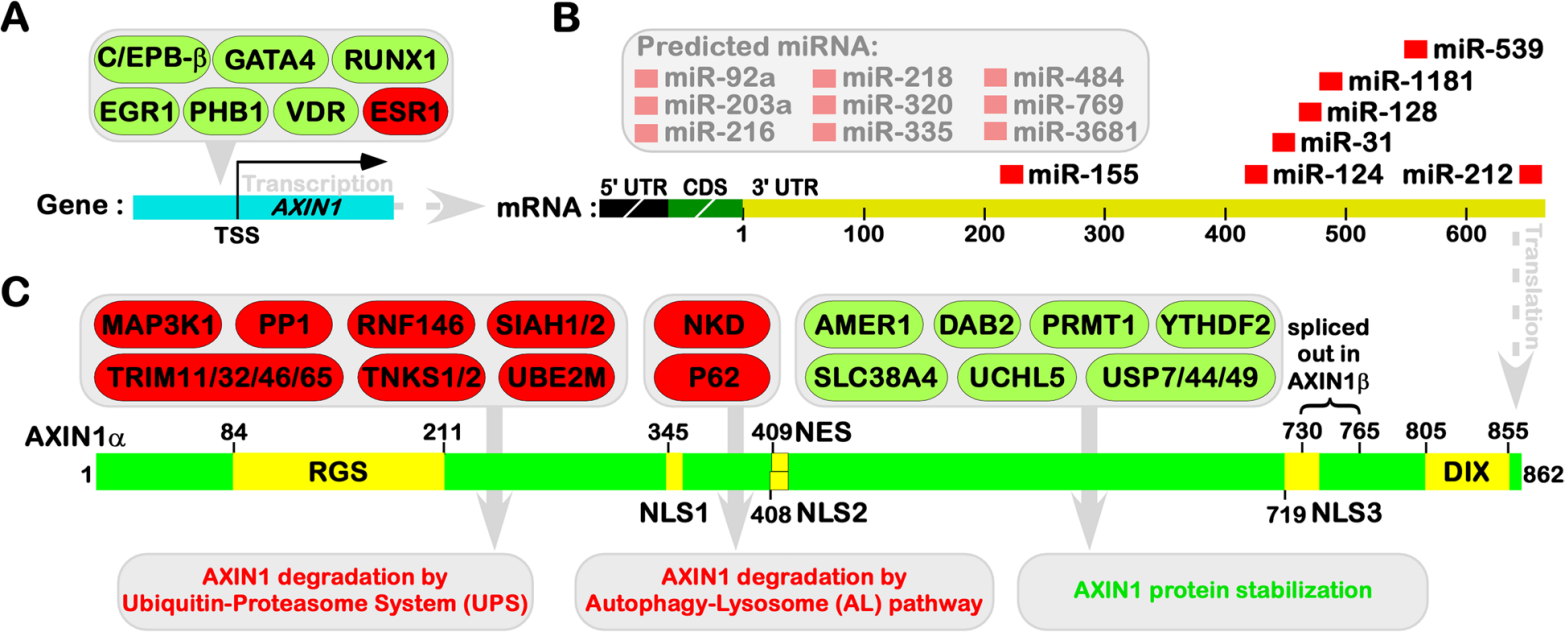
Regulation of AXIN1 expression. **A** Transcription factors regulating AXIN1 gene expression. Activating factors are denoted with a green background, while inhibitory factors are marked with a red background. **B** Micro-RNA regulators of AXIN1 mRNA. Grey boxes represent unverified targets. **C** Protein-level regulators of AXIN1. Proteins promoting AXIN1 stability are indicated with a green background, while proteins promoting AXIN1 degradation are marked with a red background

Investigations into the transcriptional regulation of the *AXIN1* gene have unveiled a myriad of transcription factors that actively engaged in promoting its transcriptional activation. These factors include GATA4 [13], RUNX1 [14], VDR [15], C/EPB-β [16], PHB1 [17], and EGR1 [18]. Intriguingly, the transcriptional activation of *AXIN1* can be modulated through competitive inhibition exerted by ERα/ESR1, which counters the activities of GATA4 and RUNX1 [13, 14] (Fig. 3A). Beyond the influence of transcription factors, epigenetic modifications assume a pivotal role in gene expression regulation. Notably, X-ray irradiation has been documented to upregulate *AXIN1* expression via genomic DNA demethylation. Distinct methylation statuses of the *AXIN1* gene in lung cancer cells correlate with varying radio-sensitivities, highlighting the influence of methylation modifications on *AXIN1* transcriptional levels [19]. Furthermore, X-ray irradiation induces an elevation in *AXIN1* expression and triggers apoptosis in lung cancer cells through the inhibition of histone deacetylase HDAC1/2 [20], which potentially constituting one of the mechanisms underlying radiotherapy-induced tumor suppression. Additionally, studies have reported that treatment with cyclosporine A diminish the transcription levels of *AXIN1* in gingival tissue. Activated WNT/β-catenin signaling is concurrently associated with gingival overgrowth, although the precise underlying mechanism remains enshrouded in ambiguity [21].

The degradation of mRNA orchestrated by microRNAs (miRNAs) represents a pivotal avenue for post-transcriptional gene regulation. A multitude of miRNA binding sites are evident within the 3' untranslated region (3'UTR) of human *AXIN1* mRNA, affirming the presence of widespread post-transcriptional regulation of *AXIN1* gene expression (Fig. 3B). Notably, miR-155 targets the 221-228 bp region within the 3'UTR of *AXIN1* [22], while Hsa-miR-124-3p.1 engages with the 433-439 bp region [23–25]. Additionally, miR-31-5p associates with the 454-460 bp region [26, 27], hsa-miR-128-3p interacts with the 464-470 bp region [28–30], miR-1181 targets the 477-483 bp region [31], and miR-212 establishes a connection with the 666-671 bp region of *AXIN1*'s 3'UTR [32]. In rats, miR-3574 has been identified binding to the 516-523 bp region of the 3'UTR of *AXIN1*, a conservation observed in humans [33]. Additionally, in rats, miR-539 has been found to interact with the 558-567 bp region of *AXIN1*'s 3'UTR [34]. Beyond these well-established miRNAs, it is worth noting that hsa-miR-216a-3p targets the 464-470 bp region, hsa-miR-3681-3p engages with the 464-470 bp region, and hsa-miR-203a-3p.1 binds to the 558-564 bp region within the 3'UTR (as predicted by the TargetScan website). Furthermore, the Mirtarbase website predicts that miRNAs like hsa-miR-335-5p, hsa-miR-320c, hsa-miR-769-3p, hsa-miR-484, hsa-miR-320a, hsa-miR-92a-3p, and hsa-miR-218-5p may also be implicated in binding to the 3'UTR region of *AXIN1*.

### Structural characteristics and turnover regulation of AXIN1 protein

The complete human AXIN1 (AXIN1α) protein encompasses 862 amino acid residues (aa) (Fig. 3C) and is encoded by the NM_003502.4 transcript. A more prevalent protein isoform, AXIN1β, is translated from the splice variant NM_181050.3 (Fig. 1B, Table 1), which lacks exon 9, resulting in the omission of 36 aa within the region aa 730–765 (YVQEVMRRGRACVRPACAPVLHVVPAVSDMELSETE) when compared to AXIN1α [1]. Comparative analysis of protein sequences across various species reveals that AXIN1 comprises two conserved structural domains. The first is the regulator of G-protein signaling domain (RGS, aa 84–211) located at the N-terminus of the AXIN1 protein. This region demonstrates a high degree of homology with regulators of G protein signaling and is a notably conserved segment within the G protein signaling regulatory factor family [1, 35, 36]. The second is the Dishevelled (DVL) and AXIN1 binding domain (DIX, aa 805–855) situated at the C-terminus of the AXIN1 protein. This domain shares homology with the DVL protein and plays an integral role in the formation of homo- or hetero-dimers involving AXIN1 [1, 37, 38].

The AXIN1 protein sequence contains both nuclear export signal (NES) and nuclear localization signal (NLS) elements. Specifically, the sequence spanning aa 409–418 (LEERLKRVRM) in AXIN1 comprises its NES sequence [1, 39, 40]. AXIN1 possesses three distinct NLS sequences, located at aa 345–354 (PYRIRKQHRR), aa 408–418 (KLEERLKRVRM), and aa 719–739 (KRASRAPSKQRYVQEVMRRGR) [1, 39, 40]. The coexistence of NLS and NES suggests that the subcellular localization of the non-membrane protein AXIN1 is considerably influenced by its binding partners. For instance, when the WNT/β-catenin signaling pathway is deactivated, AXIN1, APC, GSK3β, and CK1α assemble into the destruction complex. This complex is found in both the cytoplasm and the nucleus as punctate structures [41]. Upon binding with DVL and LPR5/6 proteins, AXIN1 localizes to the cell membrane [37, 42–44]. AXIN1 can also translocate to the nucleus when forming a complex with DAXX, HIPK2, and TP53 [45, 46]. AXIN1 is also found on the surface of the lysosome membrane when it is linked to the v-ATPase and Ragulator complexes [5]. Conversely, its interaction with γ-tubulin and PLK1 proteins results in localization at the centrosome [47–49]. Studies have additionally documented the presence of AXIN1 in mitochondria, where it exerts a suppressive effect on mitochondrial ATP synthesis [50].

An intriguing observation pertains to AXIN1β, where the deletion of 36 amino acid residues within the 730–765 leads to the loss of the third NLS observed in AXIN1α. However, a potential novel NLS signal spanning from aa 719–735 (KRASRAPSKQRTRSQRK) may be generated in AXIN1β. Also, the possible phosphorylation regulation of residues T730 and S732 in this putative third NLS in AXIN1β makes things more complicated. This suggests that different phosphorylation regulation may make AXIN1α different from AXIN1β. Nonetheless, these hypotheses necessitate empirical validation. Additionally, it is worth noting that the aa 730–765 region within AXIN1α is implicated in interactions with various proteins, including but not limited to C9orf140 [51], RNF111 [52], HIPK2 [45], and MEKK4 [53]. These interactions may potentially contribute to distinctions in the functional roles played by AXIN1β and AXIN1α.

Within the cellular landscape, protein degradation is primarily orchestrated through the ubiquitin–proteasome system (UPS) and autophagy-lysosome pathways. Remarkably, both of these pathways play a pivotal role in mediating the degradation of AXIN1 (Fig. 3C). Investigations have unearthed several key players in the UPS pathway, which directly interact with AXIN1 to facilitate its ubiquitination and subsequent degradation. These include TRIM11 [54, 55], TRIM32 [56], TRIM46 [57], TRIM65 [58], MAP3K1 [59], SIAH1/2 [60], and UBE2M [61]. AXIN1 is subject to other modifications that also influence its ubiquitination and degradation. For instance, TNKS1/2 interacts with AXIN1 to introduce PARsylation modifications within the N-terminal domain of AXIN1 [62]. Subsequently, RNF146 mediates ubiquitination and the ensuing degradation of AXIN1 [63, 64]. Additionally, the protein phosphatase 1 (PP1) can engage with AXIN1, leading to its dephosphorylation and subsequent degradation [65, 66]. AXIN1 degradation through the autophagy-lysosome pathway has also garnered attention in the research sphere. Studies have revealed that AXIN1 can connect with the HisC structure of NKD, breaking it degradation through the P62-mediated autophagy-lysosome pathway [67]. Furthermore, instances such as starvation have shown to intensify the ubiquitination modification of AXIN1, with the subsequent degradation of AXIN1 facilitated through the autophagy-lysosome pathway upon its binding with P62 [68].

Conversely, several studies have delineated a group of proteins that function to stabilize AXIN1 (Fig. 3C), thus exerting inhibitory effects on the WNT/β-catenin signaling pathway. These stabilizing influences have been documented through various mechanisms. Reports have indicated that deubiquitinating enzymes, including USP7 [69], USP44 [70], USP49 [71], and UCHL5 [72], can reduce the ubiquitination of AXIN1, thereby hampering WNT/β-catenin signaling. DAB2 contributes to an increase in the protein level of AXIN1 by impeding the dephosphorylation of PP1 on AXIN1 [73, 74]. Methylation modifications have been identified as enhancing the stability of the AXIN1 protein. For instance, protein arginine methyltransferase 1 directly interacts with AXIN1, methylating the 378th arginine residue. This modification results in increased stability of AXIN1 and concurrent inhibition of WNT/β-catenin signaling [75]. YTHDF2 [76] and SLC38A4 [77] have also been reported as contributors to AXIN1 stabilization, thereby impeding its degradation. AMER1 has been noted for its negative regulatory role in WNT/β-catenin signaling. It elevates the stability of AXIN1 at the plasma membrane via funcitioning as a scaffold protein for the β-catenin destruction complex [78]. It is essential to recognize that these studies have been conducted in specific tumor types or cell contexts, and as such, some regulatory mechanisms may be influenced by the genetic backgrounds in which they occur. Nevertheless, the fundamental insights into gene expression regulation and protein–protein interactions can be regarded as general and foundational attributes of AXIN1.

### The interaction network and signaling pathway involved in AXIN1

AXIN1 was first found to negatively regulate axis formation during embryonic development. It has been discovered to play vital roles in various important signaling pathways related to development, metabolism, and cancer. For instant, the WNT/β-catenin signaling, Hippo signaling, TGFβ signaling, AMPK signaling, mTOR signaling, TP53 signaling, SAPK/JNK signaling, antioxidant signaling, STING signaling, and more.

#### AXIN1 regulatory control on WNT/β-catenin signaling

**Fig. 4:**
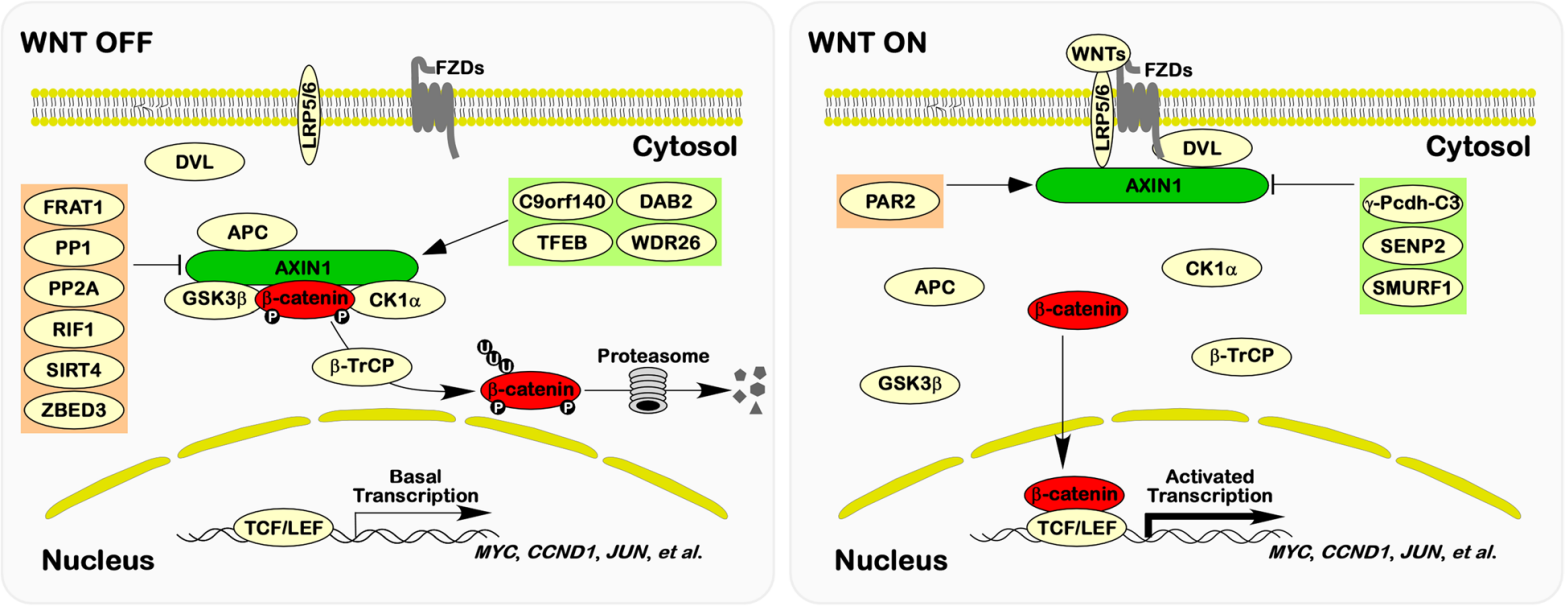
AXIN1 regulatory control on WNT/β-catenin Signaling. During WNT inactivity (WNT OFF), the homeostasis of β-catenin relies on the intricacies of the destruction complex, governing its proteasomal degradation through UPS pathway. Multiple factors, including FRAT1, PP1, PP2A, RIF1, SIRT4, and ZBED3, exert their influence on AXIN1 to inhibit the function of the destruction complex. Additionally, C9orf140, DAB2, TFEB, and WDR26 act on AXIN1 to facilitate the functioning of the destruction complex. When the WNT pathway is activated (WNT ON), AXIN1 is recruited to the WNTs-LRP5/6-FZDs-DVL complex, thereby relieving the suppression of the destruction complex on β-catenin. PAR2 serves to inhibit this process by acting on AXIN1, while γ-Pcdh-C3, SENP2, and SMURF1 also contribute to the inhibition of this process through interactions with AXIN1

The WNT/β-catenin signaling pathway is a key regulator of embryonic development and is also linked to the onset and progression of various cancers [79]. This intricate signaling network orchestrates the transmission of extracellular signals to the nucleus, although the full complement of proteins directly involved may not yet be fully elucidated. Central to classical WNT/β-catenin signaling is the meticulous regulation of the transcription factor β-catenin's activity (Fig. 4). In the absence of WNTs, β-catenin resides in the cytoplasm, subject to degradation facilitated by the destruction complex. CK1α and GSK3β sequentially phosphorylate β-catenin, triggering its ubiquitination by β-TrCP and subsequent degradation via the ubiquitin–proteasome pathway [80–84]. Conversely, in the presence of WNTs, WNTs bind to the FZDs-LRP5/6 complex located on the cell surface. WNTs stimulation induces LRP6 phosphorylation and aggregation, thereby enabling the intracellular segment of FZDs to bind with the DVL protein. This interaction results in the recruitment of the destruction complex to the cellular membrane, effectively alleviating its inhibitory influence on β-catenin within the cytoplasm. Subsequently, more β-catenin translocates into nucleus, engaging with TCF/LEF to form a complex that assumes its role as a transcription factor [85, 86].

Direct interactions involving AXIN1 and a multitude of proteins serve as the primary avenue through which cells modulate the WNT/β-catenin signaling pathway. Notably, distinctions in the composition of the AXIN1 complex are instrumental in regulating WNT/β-catenin signaling. At the core of these interactions is the AXIN1-APC-GSK3β-CK1α destruction complex, which promotes the degradation of β-catenin. Upon activation of the WNT/β-catenin signaling pathway, DVL associates with AXIN1, thereby relieving the inhibitory effects of the destruction complex on β-catenin [37, 42]. However, the interaction between SENP2/AXAM and AXIN1 inhibits the binding of DVL to AXIN1, consequently suppressing WNT/β-catenin signaling [87, 88]. The VCD structure of γ-Pcdh-C3 competes with the DVL for the DIX domain of AXIN1, thereby inhibiting the transmission of WNT/β-catenin signaling [89]. LRP5/6 serves as a pivotal actor in initiating WNT/β-catenin signal transduction [90, 91]. LRP5/6 interacts with AXIN1 and inhibits the phosphorylation of β-catenin by GSK3β [43, 44]. In a contrasting role, the interaction between PAR2 and LRP6 promotes the phosphorylation of LRP6, resulting in the recruitment of AXIN1 and activation of WNT/β-catenin signaling [92]. In contrast, SMURF1 interacts with AXIN1, facilitating its ubiquitination without leading to AXIN1 degradation. This interaction prevents AXIN1 from binding to LRP5/6, thereby inhibiting WNT/β-catenin signaling [93]. The factor GID8 has been identified as a key role in sustaining WNT/β-catenin signaling [94]. In the resting state of WNT/β-catenin signaling, AXIN1 interacts with GID8 to promote the ubiquitination and degradation of GID8. Conversely, when WNT/β-catenin signaling is activated, GID8 translocates to the nucleus, where it binds and retains β-catenin protein. Similarly, the transcription factor SP1 also plays a role in WNT/β-catenin signaling regulation [95]. The interaction between AXIN1 and SP1 leads to SP1's degradation through phosphorylation by GSK3 and ubiquitination by β-TrCP. When WNT/β-catenin signaling is activated, SP1 and β-catenin form a complex and bind together to the promoter of target genes, thereby promoting their transcription.

Recent research has shed light on the essential role of AXIN1 acetylation in the functioning of the destruction complex. Constitutive acetylation of AXIN1 is imperative for the proper operation of the complex. However, during the activation of the WNT/β-catenin signaling pathway, AXIN1 undergoes deacetylation at Lys147. This deacetylation is mediated by SIRT4 [96]. The stability of the destruction complex can also be regulated by the phosphorylation modification of AXIN1. CK1α and GSK3β phosphorylate and modify the AXIN1 protein [97–99]. Conversely, protein phosphatase 1 (PP1) engages with AXIN1 and dephosphorylates it, thereby marking it for degradation. This process weakens the functionality of the destruction complex, ultimately leading to WNT/β-catenin signaling activation [65]. In non-small-cell lung cancer (NSCLC), RIF1 has been found to activate WNT/β-catenin signaling by promoting the interaction between PP1 and AXIN1 [66]. Conversely, DAB2 acts as an inhibitor of AXIN1 dephosphorylation by PP1, thereby hindering WNT/β-catenin signaling [73, 74]. Further contributing to the complexity of WNT/β-catenin signaling regulation, WDR26, an AXIN1-interacting protein, promotes the ubiquitination modification of β-catenin [100]. PPP2CA, the catalytic subunit of PP2A, interacts with the aa 502–706 region of AXIN1 and prevents GSK3β or CK1α from destruction complex. Consequently, this leads to the dephosphorylation and stabilization of β-catenin, resulting in the activation of WNT/β-catenin signaling [101, 102]. Similarly, FRAT1 can activate WNT/β-catenin signaling by impeding the interaction between GSK3β and AXIN1 [103]. Furthermore, *C9orf140*, a downstream target gene of β-catenin, plays a role in WNT/β-catenin signaling regulation. The aa 1–239 region of the C9orf140 protein interacts with the aa 502–740 region of AXIN1. This interaction competitively inhibits the binding of PPP2CA and AXIN1, thereby promoting the degradation of β-catenin to exert a negative regulatory impact on WNT/β-catenin signaling [51]. ZBED3, another AXIN1-binding protein, interacts with GSK3β and CK1α, ultimately intensifying the interaction between ZBED3 and AXIN1. This process results in the activation of WNT/β-catenin signaling [104]. Moreover, TFEB has been identified as an AXIN1-interacting protein and is sequestered in the cytoplasm by the destruction complex. When WNT/β-catenin signaling is activated, TNKS1 performs a PARsylation modification on TFEB, facilitating its dissociation from the destruction complex. This allows TFEB to translocate to the nucleus, where it forms a complex with β-catenin and TCF/LEF1, thereby promoting the transcription of specific downstream genes within the WNT/β-catenin signaling pathway [105]. The diverse array of interacting proteins with AXIN1 contributes to the intricate regulation of WNT/β-catenin signaling. As a result, the activation or inhibition of WNT/β-catenin signaling emerges as the final outcome of the intricate interplay between multiple factors within the cell. This nuanced understanding elucidates the varying sensitivities of WNT/β-catenin signaling to WNTs under different genetic backgrounds and the distinctions between classical and non-classical pathways of WNT/β-catenin signaling.

#### AXIN1 regulatory control on Hippo and TGFβ signalings

**Fig. 5:**
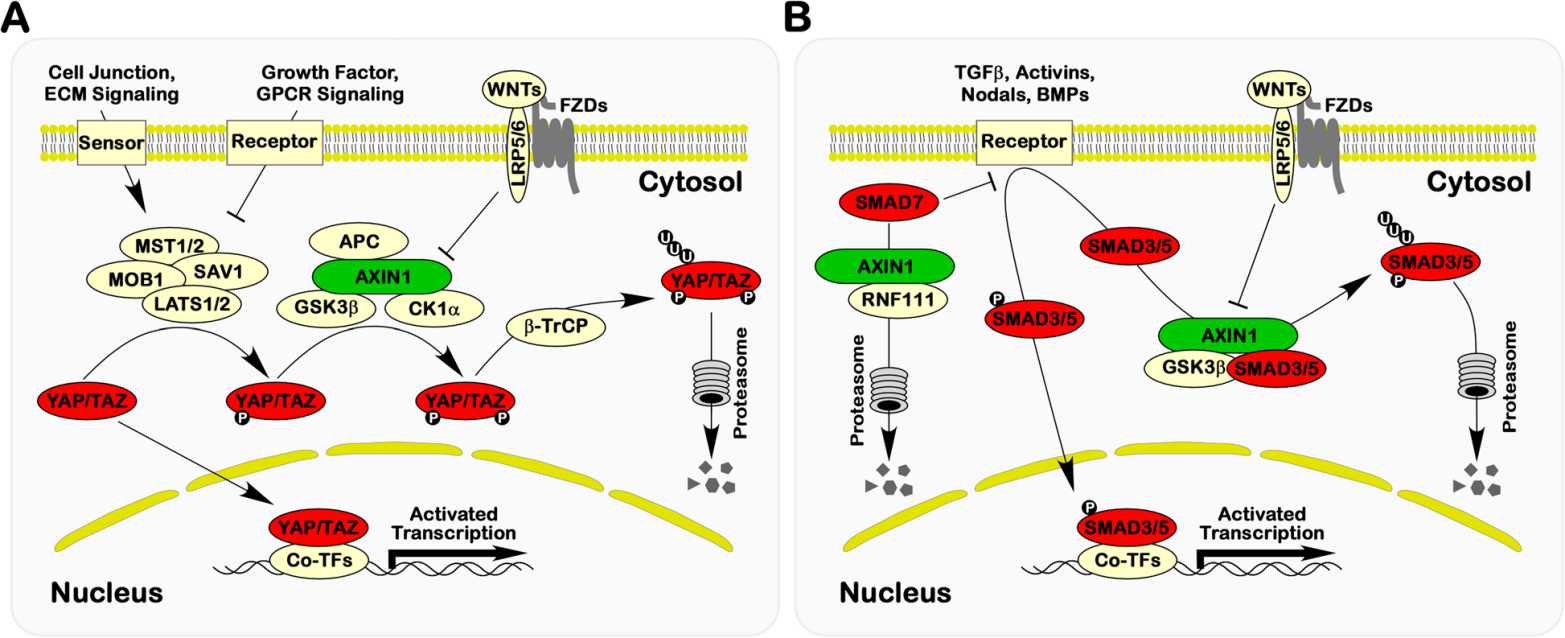
AXIN1 regulatory control on Hippo and TGF-β Signaling. **A** Within the Hippo signaling cascade, the destruction complex undergoes phosphorylation catalyzed by LATS1/2, initiating a cascading series of post-translational modifications culminating in the ultimate degradation of YAP/TAZ. This process is facilitated through the UPS pathway. **B** In the context of TGF-β signaling, AXIN1 emerges as a pivotal actor, engaging directly with SMAD3/5/7. This interaction serves as the impetus for the degradation of SMAD proteins via the UPS pathway

The Hippo pathway, highly conserved in mammals, plays a pivotal role in controlling organ size, differentiation, and tissue homeostasis [106]. Notably, despite their functional antagonism, both the Hippo and WNT/β-catenin signaling pathways share a strikingly similar architectural framework. At the core of the Hippo signaling pathway lies the regulation of the transcription factors YAP/TAZ (Fig. 5A). Activation of the Hippo pathway results in the formation of a kinase cascade involving MST1/2, SAV1, MOB1, and LATS1/2, leading to the phosphorylation and inhibition of YAP/TAZ [106]. Intriguingly, following LATS1/2-mediated phosphorylation, YAP/TAZ undergo an additional phosphorylation by the destruction complex, which subsequently ushers their degradation through the ubiquitin–proteasome pathway [2]. On the other hand, the binding of YAP/TAZ to the destruction complex promotes the ubiquitination and degradation of β-catenin, and the activation of WNT/β-catenin signaling concurrently influences both β-catenin and YAP/TAZ [107–109]. Intriguingly, research on polycystic kidney disease has revealed that TAZ interacts with AXIN1, leading to the competitive release of β-catenin from the destruction complex [110]. YAP/TAZ can indeed facilitate the dissociation of β-catenin from the destruction complex. The ultimate fate of β-catenin, whether it undergoes degradation or translocates to the nucleus, may be contingent upon phosphorylation disparities. Nevertheless, the regulation of the destruction complex on both YAP/TAZ and β-catenin provides insights into the mechanism underlying AXIN1's relevance in organ size control, regeneration, and tumorigenesis.

The Transforming Growth Factor β (TGFβ) family, comprising TGFβs, bone morphogenic proteins (BMPs), activins, and Nodal, plays a pivotal role in governing embryonic development and the maintenance of adult tissue homeostasis. The fundamental transduction of these signaling pathways hinges upon the SMAD proteins [111]. AXIN1 has been identified as the primary scaffold for TGFβ signaling transduction. However, its impact on SMADs remains a topic of contention (Fig. 5B). Analogous to the regulation of β-catenin and YAP/TAZ by the destruction complex, in the absence of TGFβ, AXIN1 and GSK-3β can associate with SMAD3, leading to its degradation. Consequently, the inhibition of the destruction complex by WNT/β-catenin signaling can also trigger the activation of SMAD3 [3, 112]. In chondrocytes, TGFβ suppresses the expression of AXIN1 and AXIN2 while promoting β-catenin signaling. Paradoxically, the induction of WNT/β-catenin signaling acts as a mitigating factor in restraining TGFβ induction of Smad3 reporter genes [113]. Within the destruction complex, AXIN1 interacts with SMAD5, a downstream effector of BMP signaling, facilitating its phosphorylation by GSK3β and subsequent ubiquitin-mediated degradation, thereby suppressing BMP signaling. The activation of BMP/SMAD5 signaling coincides with the inhibition of the destruction complex when WNT/β-catenin signaling is active [114]. Moreover, AXIN1 forms a complex with RNF111 and SMAD7, facilitating the ubiquitination and degradation of SMAD7 [52]. Although AXIN1's implication in NOTCH signaling has been documented [115, 116], its precise role remains to be elucidated. Given the current state of research, AXIN1 is emerging as a key role in the integration of WNT/β-catenin signaling, Hippo signaling, and TGFβ signaling. This suggests that AXIN1 widespread involvement in embryonic development, organogenesis, and tissue homeostasis. Simultaneously, the polymorphism of AXIN1-interacting proteins underscores the multifaceted nature of the developmental biological signals regulated by AXIN1.

#### AXIN1 regulatory control on AMPK and mTOR signalings

**Fig. 6:**
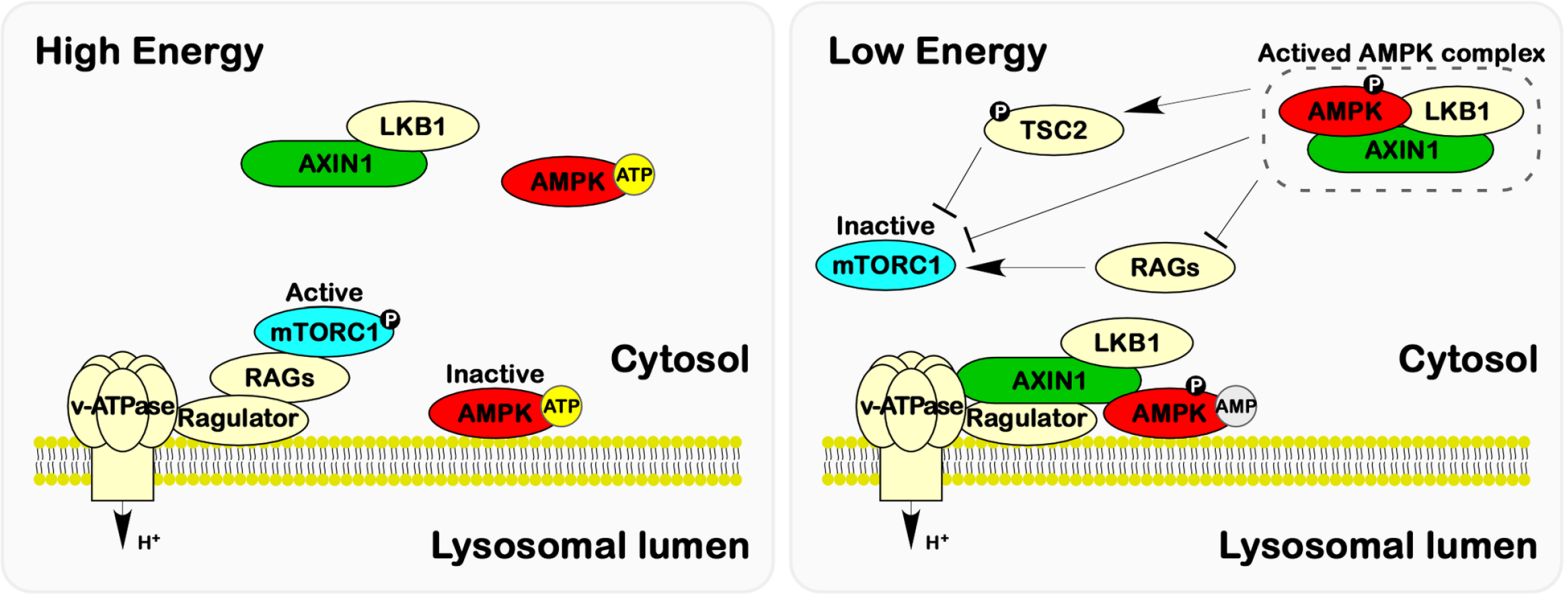
AXIN1 regulatory control on AMPK and mTOR signaling pathways. In heightened energy states, mTORC1 activation unfolds at the lysosomal membrane via the engagement of the v-ATPase-Ragulator-RAGs complex. Concomitantly, an abundance of ATP exerts inhibitory effects, delicately modulating the assembly of the AXIN1-LKB1-AMPK complex and subsequently attenuating AMPK signaling. In contrast, under conditions of energy scarcity, AXIN1 undergoes recruitment to the lysosomal membrane through the v-ATPase-Ragulator complex. This orchestrated move directly quells mTORC1 signaling. Concurrently, elevated AMP levels stimulate the assembly of the AXIN1-LKB1-AMPK complex, thereby igniting AMPK signaling

The AMPK (AMP-activated protein kinase) signaling pathway constitutes a central mechanism for sensing and regulating intracellular energy metabolism homeostasis. Under conditions of nutrient scarcity, AMPK detects increased AMP levels, subsequently undergoing phosphorylation and modification by LKB1 (Serine/Threonine Kinase 11, STK11), resulting in the activation of catabolic processes and the inhibition of anabolic pathways [117]. mTOR, a serine/threonine kinase, serves as a key regulator of cellular metabolism and facilitates cell growth in response to environmental cues. In the presence of abundant nutrients, mTORC1 (mTOR complex 1) is recruited to the lysosome through a series of events triggered by the recognition of the glycolytic intermediate, fructose-1,6-bisphosphate (FBP), by aldolase. Subsequently, mTORC1 is activated, thereby instigating anabolic pathways [5, 118, 119]. AXIN1 has emerged as a crucial mediator in the regulation of both AMPK and mTOR signaling (Fig. 6). The activation of AMPK through phosphorylation by LKB1, prompted by elevated AMP levels, critically relies on AXIN1's role as a scaffold protein. AXIN1 facilitates the assembly of a complex involving AMPK and LKB1 [4]. Further investigations have illuminated that the activation of AMPK signaling is intrinsically linked to the localization of the AXIN1/LKB1/AMPK complex on the surface of lysosomes. During periods of glucose deprivation, AXIN1 forms interactions with the v-ATPase/Ragulator complex situated on the lysosomal surface, thereby initiating the activation of AMPK signaling [5]. Meanwhile, glucose deprivation leads to the v-ATPase/Ragulator/AXIN1 complex, inhibiting the guanine nucleotide exchange factor activity of Ragulator towards RAG proteins, prompting their dissociation from the lysosomal surface and resulting in the deactivation of mTORC1 [5, 118, 119]. In this capacity, AXIN1 plays a pivotal role in orchestrating cellular synthesis and catabolism in response to fluctuations in energy status.

#### AXIN1 regulatory control on other signaling pathways

As a component of the MAPK signaling cascade, the stress-activated protein kinase (SAPK)/Jun N-terminal kinase (JNK) signaling pathway becomes activated in response to environmental stressors, inflammatory cytokines, growth factors, and agonists of G protein-coupled receptors [120]. This pathway functions by transmitting phosphorylation cascades to downstream transcription factors via JNK. Recent research has unveiled that the aa 217–352 region of AXIN1 binds to MEKK1, thereby facilitating SAPK/JNK activation [121, 122]. Furthermore, it has been observed that AXIN1 dimers, rather than monomers, can engage with MEKK1 to trigger SAPK/JNK signaling. Of note, JNK activation in heterodimers formed by AXIN1 and DVL relies on DVL itself rather than AXIN1 [123]. Additionally, the aa 678–712 region of AXIN1 can associate with MEKK4, contributing to SAPK/JNK pathway activation [53]. Research has also shown that the aa 389–473 region of CCD1 interacts with the aa 600–700 region of AXIN1, hindering the interaction between MEKK1 and AXIN1. Furthermore, CCD1's binding to MEKK4 can impede the association between MEKK4 and AXIN1, thereby suppressing the SAPK/JNK signaling pathway [124]. T Protein interaction studies have firmly established the interactions of both CK1α and CK1ε with AXIN1, while CK2α does not form a complex with AXIN1. Importantly, only CK1ε can inhibit AXIN1-mediated JNK activity [125, 126]. Intriguingly, the SUMO-1 modification of AXIN1's C-terminal region appears crucial for JNK activation but does not affect the WNT/β-catenin signaling pathway [127].

TP53 is a pivotal factor responsible for regulating the cell cycle. When AXIN1 forms a complex with DAXX, HIPK2, and TP53 (with AXIN1's aa 678–753 region interacting with HIPK2's aa 935–1050 region, AXIN1's aa 507–608 region interacting with DAXX's aa 1–197 region, and AXIN1's aa 210–337 region interacting with TP53's aa 237–289 region), the kinase activity of HIPK2 is activated. HIPK2 subsequently phosphorylates TP53 at Ser46, thereby activating TP53's function as a transcription factor [45, 46]. Notably, AXIN1 plays a critical role in determining cell fate following DNA damage by regulating the activation threshold of TP53. Upon exposure to sublethal doses of UV radiation, the interaction of PIRH2 with AXIN1 triggers cell-cycle arrest by counteracting the TP53 phosphorylation at Ser46 induced by HIPK2. In cases of lethal treatment, TIP60 disrupts the binding between PIRH2 and AXIN1, resulting in the formation of an AXIN1-TIP60-HIPK2-TP53 complex. This complex allows for maximal activation of p53, leading to the initiation of apoptosis [128]. Another well-known transcription factor, MYC, is an oncogene due to its overactivation in up to 70% of tumors. AXIN1 also functions as an inhibitor of MYC. Research suggests that AXIN1 can assemble a MYC degradation complex with GSK3β, PP2A-B56α, and PIN1. This complex promotes the ubiquitination and subsequent degradation of the MYC protein [129].

NFE2L2 is a prominent member of the CNC-bZIP antioxidant transcription factor family. NFE2L2 plays a pivotal role in the regulation of numerous genes associated with redox homeostasis by binding to ARE elements, enabling it to respond to fluctuations in redox homeostasis [130]. Recent research has revealed that AXIN1 can interact with NFE2L2 and facilitate its ubiquitination and subsequent degradation [131]. Although direct evidence is currently lacking, it is conceivable that AXIN1-mediated ubiquitination and degradation of NFE2L2 may also depend on GSK3β, based on earlier findings identifying NFE2L2 as a substrate of GSK3β [132]. This research suggests a potential role for AXIN1 in the regulation of cellular oxidative stress homeostasis. Furthermore, investigations have demonstrated that NFE2L1, another member of the CNC-bZIP family, can directly bind to the AMPK protein [133]. Considering the structural similarity among CNC-bZIP family member proteins, it is plausible that AXIN1 may engage in interactions with other family members such as NFE2L1 and NFE2L3.

Research has established a connection between AXIN1 and STING, suggesting that, during metformin treatment, AXIN1 binds to STING, preventing its ubiquitination and degradation. This interaction enhances the tumor-killing effect of T cells induced by metformin [134]. The direct regulatory role of AXIN1 on STING implies its potential involvement in cGAS/STING signaling, which is responsible for antiviral, anti-tumor, and inflammatory responses. In the context of antiviral responses, studies have demonstrated a reduction in AXIN1 levels during influenza virus infection. Overexpression or stabilization of AXIN1 has been shown to inhibit the replication of various viruses, including influenza virus, respiratory syncytial virus, and vesicular stomatitis virus [135]. However, in the case of human cytomegalovirus (HCMV) infection, AXIN1 levels increase within host cells [136]. AXIN1 has also been observed to inhibit autophagy in L929 cells induced by HSV infection, ultimately promoting virus replication [137]. The differing responses and roles of AXIN1 in viral infections are likely influenced by factors such as the specific virus type and the characteristics of host cells. Additionally, there are reports of AXIN1 interacting with Caveolin1 and regulating the LPS-induced inflammatory response in AT-I cells [138]. However, whether cGAS/STING signaling is involved in this process remains to be explored.

Indeed, AXIN1 functions primarily as a scaffold protein, playing a pivotal role in facilitating protein–protein interactions and organizing signaling complexes. In this capacity, it brings together specific kinases and their substrate proteins, enabling precise phosphorylation and regulation of target proteins within diverse signaling pathways. A prominent example of this function is seen in the destruction complex, where AXIN1 coordinates the phosphorylation of key substrate proteins, including β-catenin, YAP/TAZ, and SMADs, by kinases such as GSK3β and CK1α. AXIN1's unique ability to interact with a diverse array of partner proteins confers distinct regulatory functions across various signaling pathways. This versatility implies the potential for designing polypeptides or small molecules that target specific binding sites on AXIN1, thereby inhibiting interactions between AXIN1 and specific partner proteins. This approach could be a valuable strategy for fine-tuning or modulating specific signaling pathways, offering potential therapeutic applications. However, such targeted interventions require a deep understanding of the molecular interactions and the development of specific inhibitors, making it an area of potential interest for future research and drug discovery efforts.

### Physiological processes and pathological phenotypes involved in AXIN1

The growing body of research surrounding AXIN1 has revealed its extensive involvement in various physiological processes, highlighting its role as a critical regulator in multiple signaling networks. AXIN1 plays a key role in processes such as development, metabolism, tumorigenesis, and stress responses, among others. Dysregulation or mutations in AXIN1 can have profound consequences and may contribute to the development of a range of diseases. It's important to note that AXIN1's functions can be complex and polymorphic due to several factors, such as functional redundancy with AXIN2, tissue-specific expression patterns, and the presence of various splicing variants. These complexities make it essential for researchers to further investigate and understand the nuanced roles of AXIN1 in specific cellular contexts and different signaling pathways. As our understanding of AXIN1 continues to evolve, it may offer new insights into disease mechanisms and potential therapeutic targets for a wide array of health conditions.

### The role of AXIN1 in embryonic axis formation

The *AXIN1* gene was originally identified through its association with the *Fused* mouse phenotype, and its initial characterization revealed its pivotal role as a suppressor of embryonic axis formation by negatively modulating the WNT/β-catenin signaling pathway [1]. In *Xenopus* embryos lacking axin, abnormal gastrulation occurs, resulting in a distinctive phenotype characterized by an excess of notochord and head structures, alongside reduced tail and ventral components. This aberrant dorso-anterior development can be rescued by introducing axin mRNA into axin-deficient oocytes at the vegetal pole before fertilization [139]. Intriguingly, the phenotype is ameliorated upon ventral injection of axin mRNA at the 4-cell stage, while dorsal injection into wild-type embryos leads to ventralization, underscoring the crucial role of axin in dorso-anterior patterning [139]. In essence, AXIN1, as a central regulator of WNT/β-catenin signaling, is implicated in a myriad of developmental and regenerative processes governed by this signaling pathway. These encompass fundamental aspects such as cell proliferation, tissue differentiation, and organogenesis. Interestingly, the phenotypic manifestations observed in mice lacking AXIN1 are not uniform, signifying a degree of developmental fate determination [140, 141]. Evidently, certain AXIN1^Fu/Fu^ mice fail to survive in utero, whereas others do. Moreover, some heterozygous adult mice exhibit tail kinks, while others do not. Although epigenetic influences have been considered as contributory factors to this phenomenon, the divergence in phenotypes resulting from a single genetic variable underscores the intricate and multifaceted nature of AXIN1's physiological functions during development [142, 143].

Coincidentally, at the cellular level, AXIN1 also exhibits inconsistent phenotypes in regulating cell division. Proper centrosome replication is a pivotal prerequisite for the accurate progression of cell division, ensuring the faithful segregation of chromosomes and averting aneuploidy. A research investigation has elucidated the significance of the interaction between AXIN1 and γ-tubulin in fostering centrosome duplication. This partnership, however, must be finely regulated to prevent excessive centrosome production within cells. Such regulation occurs through the phosphorylation of AXIN1 at the S157 site by PLK1. This phosphorylation event effectively disengages AXIN1 from γ-tubulin, thereby mitigating the risk of excessive centrosome generation [47]. Specifically, the knockdown of the AXIN1 gene in mouse oocytes yields pronouncedly defective spindles, misaligned chromosomes, impediments in the extrusion of the first polar body, and compromised pronuclear formation [144]. These findings collectively underscore the indispensable role of AXIN1 in orchestrating spindle organization and facilitating cell cycle progression during the intricate meiotic maturation of mouse oocytes. It should be noted that the introduction of the S157A mutation in AXIN1, while preserving the continuous binding between AXIN1 and γ-tubulin, doesn't consistently result in the development of multiple centrosomes in all cells [47]. This inherent variability underscores the complex and diverse roles of AXIN1. Further research endeavors are warranted to elucidate the underlying mechanisms and interactions that underpin these intricate phenotypic variations.

### The role of AXIN1 in skeletal development

In a recent breakthrough, researchers have unveiled the manifestation of a human genetic disorder caused by AXIN1 mutations. In particular, bi-allelic variants disrupting the C-terminal DIX domain of AXIN1 were identified as causative factors for craniometadiaphyseal osteosclerosis with hip dysplasia [145]. Simultaneously, investigations in murine models have brought to the fore the pivotal role of AXIN1 in lower limb development. Specific deletion of AXIN1 within limb mesenchymal cells culminated in limb anomalies akin to fibular hemimelia (FH), typified by fibular and femoral malformations. These malformations were coupled with reduced osteoclast formation and impairments in angiogenesis, both notably mitigated through the inhibition of β-catenin or BMP signaling [114]. Further investigations revealed that the loss of AXIN1 in osteoblast precursor cells predominantly affected osteoclast formation within the metaphyseal bone region, leading to delayed bone growth in mice [146]. Additionally, specific deletion of AXIN1 in condylar chondrocytes resulted in an osteoarthritis-like phenotype in the temporomandibular joint, a degenerative disease characterized by pathological condylar cartilage degeneration in adults. This condition was attributed to the activation of WNT/β-catenin and FGF/ERK signaling pathways [147]. These findings collectively suggest that AXIN1 plays a pivotal role in orchestrating the actions of osteoblasts and osteoclasts by modulating developmental-related signaling pathways. In chondrocytes, AXINs serve to integrate signals between the WNT/β-catenin and TGFβ/SMAD pathways. The suppression of AXIN1 and AXIN2 expression by TGFβ augments WNT/β-catenin signaling, thereby expediting chondrocyte maturation [113]. Furthermore, in rat primary chondrocytes, treatment with IL-1β was found to diminish USP49 expression, an enzyme involved in deubiquitinating AXIN1. This led to AXIN1 accumulation, thereby activating WNT/β-catenin signaling and promoting the progression of osteoarthritis (OA) [71]. Similarly, UBE2M was observed to facilitate AXIN1 ubiquitination and subsequent degradation within chondrocytes, ultimately intensifying apoptosis among OA-afflicted chondrocytes, through activation of the WNT/β-catenin pathway [61]. These insights into the multifaceted role of AXIN1 in skeletal development and pathologies offer an enriched comprehension of its regulatory functions.

### The role of AXIN1 in neural system

In addition to its pivotal role in skeletal development, AXIN1 is equally indispensable for the development of various other tissues, particularly within neural and muscular systems [148]. Strikingly, AXIN1 has recently come under scrutiny in the context of schizophrenia (SCZ), revealing a sex-specific association in males. Experimentation involving the overexpression of S-SCAM within the forebrain of murine models has elucidated sex-specific impairments related to synaptic plasticity and working memory, a phenomenon predominantly manifesting in male subjects [149]. These impairments are primarily attributed to the reduction of AXIN1 due to S-SCAM, resulting in functional deficits in GSK3β, particularly affecting male subjects. Intriguingly, the administration of 17β-estradiol has demonstrated a potential therapeutic avenue by mitigating neural impairments arising from S-SCAM overexpression in female subjects. This therapeutic effect is achieved through the upregulation of AXIN2 within neurons, in conjunction with the augmentation of synaptic GSK3β levels. Moreover, elevating AXIN1 levels via the administration of the small molecule XAV939 has unveiled a dual capacity, enhancing both embryonic neurogenesis and influencing social interaction behaviors, while simultaneously fostering adult hippocampal neurogenesis and conferring an antidepressant effect [62, 150]. Additionally, investigations into Sema3A-induced axonal transport have revealed a previously uncharted role of AXIN1. Specifically, the depletion of AXIN1 expression was observed to inhibit both anterograde and retrograde axonal transport processes. This showed that AXIN1 is involved in Sema3A-induced bidirectional axonal transport dynamics [151].

In addition, a genomic convergence of locus-based GWAS meta-analysis identified AXIN1 is a Parkinson's Disease (PD) gene [152]. And miR-128 has been shown to play a protective role in safeguarding dopamine neurons and hippocampal neurons from apoptosis in the context of PD. This protective mechanism is facilitated by miR-128's targeting of AXIN1, subsequently activating WNT/β-catenin signaling pathways [29, 30]. Similarly, miR-212-3p has demonstrated the potential to mitigate the progression of PD by targeting AXIN1 [32]. In a separate study involving rats, the knockdown of AXIN2 in the context of PD has been found to activate WNT/β-catenin signaling pathways. This activation sequence, in turn, promotes mitochondrial biosynthesis and the generation of dopaminergic neurons, ultimately ameliorating the behavioral deficits associated with PD [153]. Studies in zebrafish have provided further insights into the role of axin in neural development. Mutations in axin result in atypical fate determination of the eyes and telencephalon [154], alongside disruptions in the establishment of nervous system asymmetries [155]. Significantly, it is worth noting that distinctions exist in the regulation of WNT/β-catenin signaling when comparing AXIN1 and AXIN2 within the nervous system. The findings suggest that the specific regulation of AXIN1 or AXIN2 expression, depending on the specific neurological disorder in question, may hold promise for more targeted and efficacious therapeutic interventions.

### The role of AXIN1 in muscular system

Recent research involving cardiac fibroblasts has unveiled the regulatory role of miR-124, originating from exosomes, in cardiac tissue. This miRNA suppresses the expression of AXIN1 within cardiac fibroblasts, consequently activating WNT/β-catenin signaling and thereby promoting the activation and proliferation of fibroblasts [23]. Similarly, miR-128 has been identified as a regulator of WNT/β-catenin signaling in the heart, primarily by inhibiting AXIN1 [28]. However, it's worth noting that the overexpression of miR-128 can negatively impact the proliferation of cardiomyocytes and cardiac function [156]. Chen and colleagues have reported that miR-3574 has a mitigating effect on cardiomyocyte injury induced by intermittent hypoxia in rats, a condition often encountered in sleep apnea. This protection is achieved through AXIN1 inhibition [33]. Furthermore, in the context of streptozotocin-induced type 1 diabetes (T1DM) in mice, metronidazole (MTZ) has demonstrated the capacity to ameliorate the structural and functional alterations in cardiac tissue caused by T1DM. This effect is attributed to the enhanced interaction between AXIN1 and β-catenin, ultimately leading to the inhibition of the WNT/β-catenin signaling pathway [157].

In skeletal muscle, where both AXIN1 and AXIN2 are expressed, AXIN2 plays a more prominent role, particularly in myogenesis [158–160]. In this context, AXIN2 can functionally replace AXIN1 in regulating AMP-activated protein kinase (AMPK). Notably, knocking out AXIN1 does not alter the regulation of AMPK/mTORC1 or glucose metabolism in mouse skeletal and cardiac muscle cells, highlighting the division of labor between AXIN1 and AXIN2 [161, 162]. These studies illuminate the tissue-specific roles of AXIN1 and AXIN2 in cardiac and skeletal muscle. In the nematode *Caenorhabditis elegans*, research highlights the significance of AXIN1 in muscle biology. The homologous gene of AXIN1, known as PRY1, exhibits robust expression in muscle tissues. Overexpression of PRY1 in the muscle significantly extends the organism's lifespan, retards the aging of muscle tissues, and enhances mitochondrial morphology [163]. However, it's worth noting that studies exploring the extension of lifespan by AXIN1 in vertebrates remain notably absent in the current literature.

### The role of AXIN1 in cancer

Cancer cells are characterized by several hallmarks, including sustained proliferative signaling, metabolic reprogramming, immune evasion, and beyond. AXIN1 exerts complex regulation on a variety of signaling pathways, including WNT/-catenin, AMPK, mTOR, TGF, TP53, SAPK/JNK, and antioxidant signaling, underscoring its extensive involvement in various aspects of oncogenic processes. Indeed, extensive research have illuminated the prevalence of AXIN1 mutations or dysfunction across a broad spectrum of malignancies. These include but are not limited to breast tumors [164], colorectal cancer [164, 165], hepatocellular carcinoma (HCC) [166–170], oral squamous cell carcinoma [171], NSCLC [172–174], astrocytoma [175], neuroepithelial brain tumors [176], glioblastoma [177], solid pseudopapillary neoplasm of the pancreas [178], gastric neoplasia of chief cell-predominant type [179], basal cell adenoma [180], among others. Furthermore, certain splice variants of AXIN1 may exert oncogenic effects. One example is circAXIN1, a new splice variant that codes for 295 aa that has been shown to turn on WNT/β-catenin signaling by blocking the function of full-length AXIN1 in gastric cancer tissues [10]. Moreover, findings indicate that APEX1's influence on AXIN1 transcript splicing in non-small cell lung cancer cells may serve to promote tumor progression [174]. These comprehensive observations underscore the intricate involvement of AXIN1 in the intricate landscape of cancer pathogenesis.

**Fig. 7:**
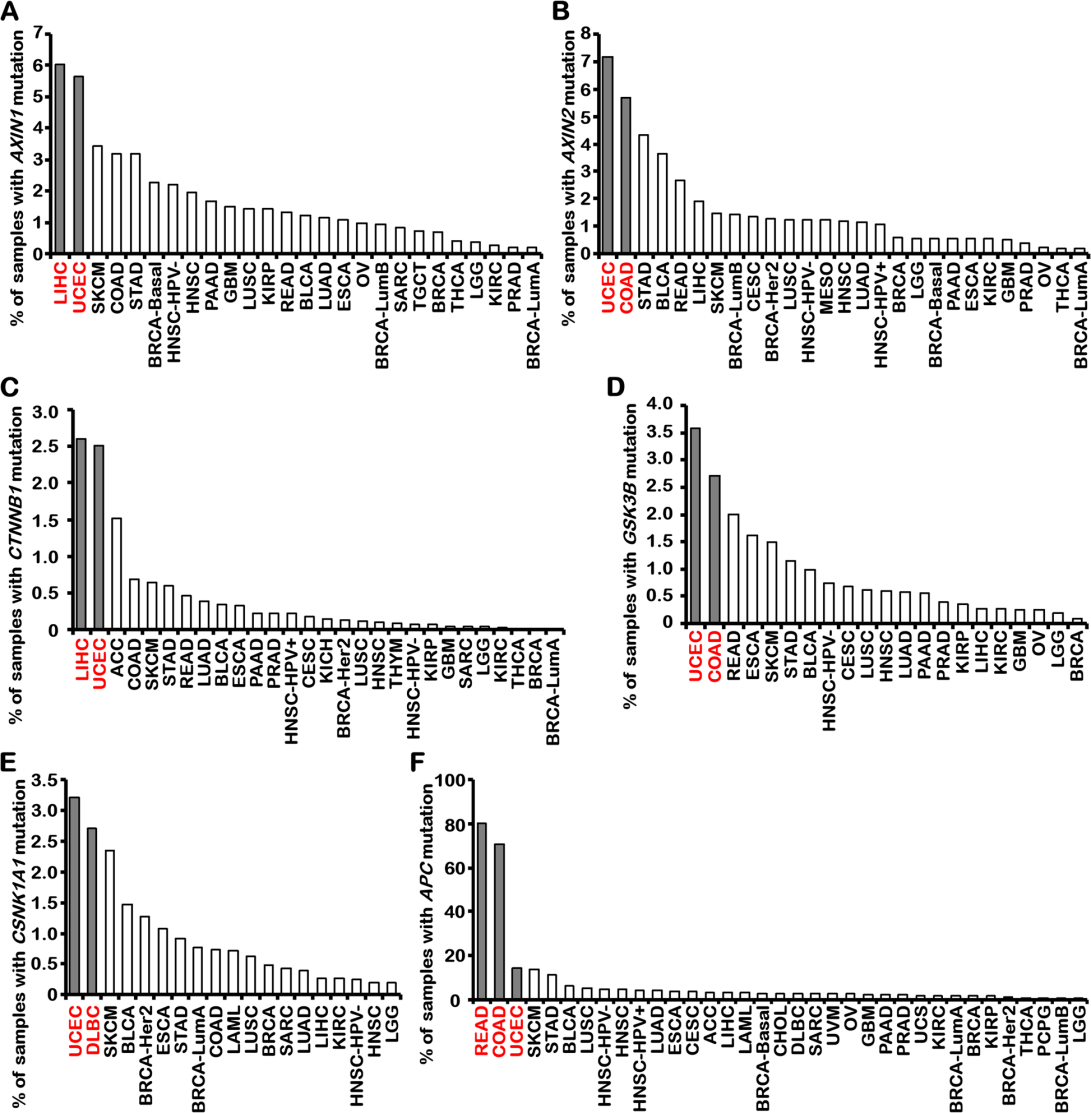
Mutation statistics of the destruction complex members in the TCGA data. Mutation statistics of *AXIN1* (**A**), *AXIN2* (**B**), *CTNNB1* (**C**), *GSK3B* (**D**), *CNSK1A1* (**E**), *APC* (**F**) in the TCGA data using the TIMER platform. (Data from Tumor IMmune Estimation Resource: http://timer.cistrome.org/)

An analysis of mutation statistics for the destruction complex members within The Cancer Genome Atlas (TCGA) dataset, conducted using the TIMER platform, has unveiled noteworthy insights. Notably, *AXIN1* exhibits the highest mutation rate in liver hepatocellular carcinoma (LIHC) at 6.03%, with uterine corpus endometrial carcinoma (UCEC) following closely behind at 5.65% (Fig. 7A). Similarly, *AXIN2* reveals the highest mutation rate in UCEC at 7.16%, with colon adenocarcinoma (COAD) showing a notable 5.67% mutation rate (Fig. 7B). Of particular note, *CTNNB1*, which encode the β-catenin protein, showcases a parallel trend, exhibiting its highest mutation rate in LIHC at 26.03% and a substantial 25.05% mutation rate in UCEC (Fig. 7C). In alignment with this pattern, *GSK3B* reveals its highest mutation rate in UCEC at 3.58%, while COAD follows with a 2.71% mutation rate (Fig. 7D). Likewise, *CSNK1A1*, which encode the CK1α protein, displays a generally low mutation rate in tumors, with its highest mutation rate in UCEC at 3.20% (Fig. 7E). On the other hand, APC mutations commonly occurs in colorectal cancer, with a scary 80% mutation rate in rectum adenocarcinoma (READ) and a substantial 70.44% mutation rate in COAD (Fig. 7F). As expected, UCEC demonstrates the third-highest mutation rate at 14.12% among the tumors. The heightened mutation rates observed in these destruction complex members across various tumor types suggest a pivotal role for the WNT/β-catenin signaling pathway. Furthermore, this underscores its significant correlation with the periodic regularity of the endometrial cycle, particularly evident in UCEC. Related to this, elevated levels of AXIN1 in plasma or serum have also been identified as a potential biomarker for endometriosis [181]. Even in cases where mutations do not directly impact protein sequence and function, the substantial mutation rates signify the indispensable anti-cancer functions these genes serve within their respective tumor types, potentially rendering them as valuable therapeutic targets.

In addition to the influence of genetic mutations leading to the loss of AXIN1 function, extensive research has delved into the role of epigenetic modifications, gene transcription, protein degradation, and other facets that impact AXIN1 function within the context of cancer research. Notably, lung cancer cells, characterized by distinct methylation statuses of the AXIN1 gene, demonstrate varying degrees of radiosensitivity. In this context, the inhibition of lung cancer cells induced by X-rays is potentially mediated through the upregulation of AXIN1. This heightened expression stems from genomic DNA demethylation and histone acetylation processes [19, 20]. In breast cancer, the transcriptional repression of AXIN1 by ERα is antagonized by RUNX1. This leads to an increase in AXIN1 expression, consequently repressing the WNT/β-catenin signaling pathway [14]. Similarly, in colorectal cancer cells, the synthetic small molecule FL3 assumes a pivotal role in blocking the phosphorylation of PHB1 at Thr258. This effect prompts the nuclear translocation of PHB1 and its subsequent binding to the Axin1 promoter. This event activates Axin1, offering a promising strategy to combat cancers that are dependent on the WNT/β-catenin pathway [17].

The post-transcriptional regulation of genes through miRNA-mediated mRNA degradation plays a vital role in gene expression control. Currently, various miRNAs have been identified as regulators that inhibit AXIN1 post-transcriptionally, thereby promoting tumor progression. Prossomariti and colleagues revealed that miR-155-5p interacts with the 3'UTR of AXIN1, leading to sustained WNT/β-catenin activation in colorectal cancer cells [22]. Yang and colleagues reported elevated expression of miR-124-3p.1 in advanced Triple-negative breast cancer (TNBC) patients, with this miRNA predicting poor overall survival in TNBC patients. The mechanism underlying its function involves the targeting of the tumor suppressor gene AXIN1, thereby activating the WNT/β-catenin signaling pathway [24]. Yu et al*.* demonstrated that upregulation of miR-31a-5p promoted the proliferation, migration, and tube formation of endothelial progenitor cells, potentially benefiting aneurysm repair. This effect was achieved by direct targeting of the 3'UTR of AXIN1 messenger RNA, leading to the repression of its expression [26]. On the other hand, decreasing microRNA-31-5p stops osteosarcoma cells from proliferation and invasion by increasing AXIN1 and suppressing the WNT/β-catenin signaling pathway [27]. In a study by Song et al., it was revealed that AXIN1 is a direct target of miR-1181, which exhibits significant overexpression in HCC tissues [31].

Factors that modulate AXIN1 at the protein level, as illustrated in Fig. 3C, theoretically possess the capacity to target AXIN1 and influence tumor progression. For instance, TRIM11 has been identified as a promoter of AXIN1 ubiquitination and degradation, thereby contributing to the progression of lymphoma [54]. TRIM65 similarly facilitates AXIN1 ubiquitination and degradation, promoting HCC progression [58]. Hunger-induced ubiquitination of AXIN1 promotes bladder cancer progression [68]. Conversely, deubiquitination modifications of AXIN1 often lead to protein stabilization, underscoring its tumor-suppressive role. USP44, for instance, reduces AXIN1 ubiquitination to inhibit colorectal cancer cell proliferation [70]. Additionally, SLC38A4 hinders liver cancer progression by preventing AXIN1 protein degradation [77]. YTHDF2, by binding to and stabilizing AXIN1 expression, enhances tumor cell resistance to chemotherapy in cervical cancer cells [76]. Research has provided insights into the potential therapeutic value of modulating AXIN1 expression. For instance, inhibiting nicotinamide phosphoribosyltransferase (NAMPT) has been shown to suppress colorectal cancer cell proliferation by increasing AXIN1 expression [182]. Beyond endogenous regulations, certain exogenous small molecule compounds have been identified for their direct or indirect effects on AXIN1. Notable examples include XAV939 [62], IWR-2/3 [183], SKL2001 [184], HLY78 [185], SEN461 [186], Lithium ion [187], FK866 [182], and Ophiopogonin B [188]. While some of the underlying mechanisms remain to be fully elucidated, these small molecules hold significant potential for the treatment of AXIN1-related diseases (Table 2).

**Table 2:** Small molecule compounds that regulate or target AXIN1

Agents	Effect	Mechanism	References
XAV939	Stabilization of AXIN1	Inhibiting TNKS1/2	[62]
IWR-2/3	Stabilization of AXIN1	Perhaps by inhibiting TNKS1/2	[183]
SKL2001	Active WNT/β-catenin	Disrupts the AXIN1/β-catenin interaction	[184]
HLY78	Active WNT/β-catenin	Potentiates the AXIN1/LRP6 interaction	[185]
SEN461	Stabilization of AXIN1	Unknown	[186]
Lithium ion	Depletion of AXIN1	Unknown	[187]
FK866	Stabilization of AXIN1	Unknown	[182]
Ophiopogonin B	Stabilization of AXIN1	Unknown	[188]

A comparative analysis of mutations in the members of the destruction complex across different tumors, as depicted in Fig. 7, highlights significant tissue-specific variations, particularly within complexes composed of AXIN1 or AXIN2. Notably, in the mouse small intestine and colon, AXIN1 deficiency does not impede intestinal differentiation, as AXIN2 can effectively compensate for the loss of AXIN1, leading to the downregulation of WNT/β-catenin activity [189]. However, the mutation data suggests that *AXIN1* and *CTNNB1* play more pivotal roles in the liver. In a tissue-based study conducted by Kim et al., it was found that 25% (9/36) of HCC tissues exhibited missense mutations in *AXIN1*, and 66.7% (24/36) exhibited reduced or absent *AXIN1* expression. Intriguingly, the mutation and dysregulation rates for *CTNNB1* were significantly lower, at 2.8% (1/36) and 30.6% (11/36), respectively, compared with *AXIN1* [167]. Consistently, no *CTNNB1* mutations were found in a study involving 168 breast cancer tissues. Higher levels of nuclear β-catenin expression correlated with lower levels of AXIN1 expression. Likewise, positive AXIN1 expression levels were lower in breast carcinomas, and AXIN1 expression inversely correlated with tumor size, histological grade, clinical tumor, node, metastasis stage, and lymph node metastasis [190]. This suggests that WNT/β-catenin signaling activation primarily promotes the proliferation of normal or cancerous cells, whereas AXIN1 deletion can initiate carcinogenesis in normal cells. The activation of WNT/β-catenin signaling during hepatocellular carcinoma initiation and development may represent an additional phenomenon following the suppression of AXIN1. This is further substantiated by evidence that liver-specific β-catenin knockout affects liver growth and regeneration [191]. Conversely, β-catenin overexpression augments liver size and regenerative capacity without inducing cancer [192]. Correspondingly, AXIN1 overexpression exerts a suppressive effect on liver cancer [167–169]. AXIN1 deficiency activates WNT/β-catenin signaling, TGFβ signaling, and YAP/TAZ, but only inhibiting YAP/TAZ can curtail hepatocellular carcinoma resulting from AXIN1 deficiency [115, 116, 193, 194]. These findings suggest that, at least in the liver, sustained WNT/β-catenin signaling activation is more likely to promote cell proliferation during carcinogenesis due to AXIN1 dysfunction, whereas Hippo signaling dysregulation may initiate cell carcinogenesis.

### Summary and future perspectives

**Fig. 8:**
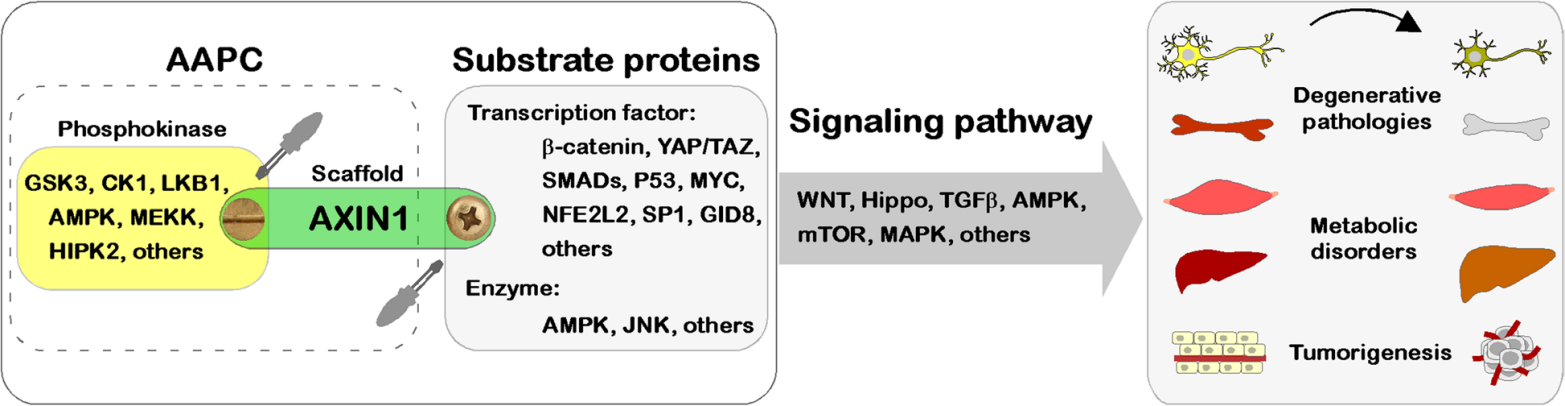
The scaffold protein AXIN1 as a promising pharmacological target

Initially, AXIN1 was recognized as a suppressor of WNT/β-catenin signaling, leading researchers to predominantly associate AXIN1's functions with WNT/β-catenin signaling in subsequent investigations, regarding the destruction complex as an integral element of the WNT/β-catenin pathway. However, as more AXIN1-interacting proteins have been unveiled, research has revealed the destruction complex as a phosphokinase complex scaffolded by AXIN1. This complex, known as the AXIN-associated phosphokinase complex (AAPC), serves as the direct conduit through which AXIN1 engages in various signaling pathways (Fig. 8). In the realm of WNT/β-catenin signaling, for instance, AXIN1 facilitates the phosphorylation of β-catenin by CK1/GSK3. Pertaining to AMPK signaling, AXIN1 orchestrates the phosphorylation of AMPK via LKB1. In the context of JNK signaling, AXIN1 contributes to the phosphorylation of JNK by MEKK, among other intricate roles. In essence, AAPC functions as an intermediary in the transfer of phosphate groups, and the acquisition of a phosphate group by a substrate triggers a paradigm shift in its fate. It's noteworthy that the transfer of phosphate groups is typically an energy-consuming process that entails ATP consumption. However, the influence of cellular energy status on AXIN1 function remains underexplored. We postulate that variances in cellular energy distribution or phosphate group donors may represent pivotal mechanisms regulating the activity of specific AAPCs.

Although AXIN1 exhibits a widespread expression pattern across various tissues without over tissue-specific distribution (Fig. 2), its functionality varies considerably in different tissue contexts. This versatility arises from AXIN1's extensive interactions with various proteins, creating intricate signaling networks. Nevertheless, AXIN1's functions maintain spatial specificity through its diverse subcellular localization patterns, ensuring that its actions remain well-ordered and do not interfere with one another. For instance, the association of AXIN1 with the Ragulator complex leads to its localization at lysosomal surfaces, where it plays a role in the regulation of AMPK and mTOR signaling. In contrast, the destruction complex predominantly localizes within the cytosol, where it exerts inhibitory control over signaling pathways relevant to development. Furthermore, when AXIN1 is found in mitochondria, it is able to suppress ATP synthesis. This spatially specific distribution of AXIN1 or the specific subcellular localization of its interacting partners guides AXIN1's participation in molecular functions. In this context, AXIN1 tends to engage more substantially in processes mediated by interacting proteins that are more abundant or exhibit higher binding affinities, rather than engaging in a balanced and uniform contribution across all relevant signaling pathways. Consequently, disparities in the abundance of AXIN1-interacting proteins or differences in the signaling pathway throughput may serve as direct determinants of the functional variances exhibited by AXIN1 across distinct tissues. Nonetheless, the challenge remains in devising strategies for achieving tissue-specific targeting and regulation of AXIN1, given its widespread expression, representing an avenue warranting further exploration.

AXIN1 plays a pivotal role in the regulation of essential cellular processes, encompassing the maintenance of cell stemness, proliferation, differentiation, and programmed cell death. This multifaceted involvement is achieved through the direct modulation of key developmental signaling pathways, such as WNT/β-catenin, Hippo, and TGFβ signaling. By doing so, AXIN1 exerts regulatory influence over critical physiological processes like embryonic development, organogenesis, and tissue homeostasis. Moreover, AXIN1 directly governs the core signaling pathways responsible for metabolic homeostasis, including AMPK and mTOR signaling, thereby influencing the cell's capacity to perceive and respond to internal and external energy cues. In addition to its role in development and metabolism, AXIN1 is integral in orchestrating cellular responses to stress, where it directly regulates signaling pathways like MAPK, STING, and NFE2L2. AXIN1's profound impact on inhibiting developmental signaling pathways and its contributions to maintaining metabolic homeostasis suggests its potential as an effective therapeutic target for conditions encompassing functional degeneration, metabolic disorders, cancer, aging, and beyond (Fig. 8). Regrettably, research within the domain of AXIN1, particularly its applied aspects, remains in its early stages. Both mechanistic investigations and applications of AXIN1 demonstrates promising but challenging avenues for further exploration.

## Data Availability

No new data were generated or analyzed in support of this review.
